# Some Remarks on the Value of Vegetable Alteratives in Chronic, Venereal, Tubercular and Malarial Diseases

**Published:** 1892-11

**Authors:** Thos. H. Manley

**Affiliations:** New York; Member of N. Y. State and County Medical Associations, American Medical Association, Visiting Surgeon to Harlem Hospital, etc.


### Selections and Abstracts.

### SOME REMARKS ON THE VALUE OF VEGETABLE ALTERATIVES IN CHRONIC, VENEREAL, TUBERCULAR AND MALARIAL DISEASES.

BY THOS. H. MANLEY, M. D., NEW YORK.
Member of N. Y. State and County Medical Associations, American Medical Association, Visiting Surgeon to Harlem Hospital, etc.

Speaking of the elements of those medicines which serve a useful purpose, in exhaustive and debilitating diseases, Dr. Manley says, that there are limitations to the province of physiological chemistry or bio-chemistry, in being able to satisfactorily explain the *modus operandi* of very many of our most valuable medicinal agents. This is particularly true of the vegetable tonics, when administered in wasting diseases, We may prescribe iron, arsenical, quinine or other salts in malarial, tubercular, or syphilitic anæmia, occasionally, in vain, when, if we place our patient on fresh infusions, decoctions or tinctures, immediate benefit will follow. The profession is not by any means in accord with those who would have us believe that the time has arrived, when medicines can be prescribed according to any set of rules, whatever scientific basis their construction may rest on.

Warburg's tincture, one of the most valuable anti-malarial remedies known, unfortunately is a secret, quack remedy, though owned by the British government. Huxam's tincture. so long a secret compound of the Birmingham chemist, is now common property of the profession.

Cod-liver oil and its many preparations have held their own well in pulmonary tuberculosis. But, there are many phases of surgical tuberculosis and those conditions of malnutrition resulting from syphilis, in which the oil is not well borne. Here the vegetable tonics, particularly those rich in the alkaline salts, are invaluable. The tincture of hops, decoction of sarsaparilla, gentian, columbo, or chamomile, either may be taken alone or in combination. A valuable combination of herb extracts was elaborated in the Southern States by members of the medical profession, as a substitute for mercury and the potassium iodide, during the late war of the rebellion, when all pharmaceutic supplies were shut out by the blockade and the advancing lines of the enemy. It is known to pharmacists and practitioners as Verrhus Clemiana, and is composed of Clematis-erecta, Prinus-verticillatus, Fraxinus Americana, Rhus-Glabrum, and one-eighth of one per cent. of Venanatic acid; all indigenous in the Southern States. I have extensively employed this compound in many cases of chronic, tubercular, glandular and bone diseases, besides other wasting maladies, with excellent results. Indeed, in these times, pharmacy yields a large number of vegetable elixirs, so palatable and easy of assimilation that one should always give them a protracted trial in the vast majority of tubercular or syphilitic, bone or joint disease, before any sort of sanguinous operation should be thought of.—*Doctor's Weekly.*

